# Value of High Frame Rate Contrast-Enhanced Ultrasound in Evaluating Vascular Morphology of Renal Cell Carcinoma

**DOI:** 10.2174/0115734056420904251202071810

**Published:** 2026-03-03

**Authors:** Jingling Wang, Wenxin Yuan, Hailan Wu, Long Gao, Weiping Zhang

**Affiliations:** 1 Departments of Ultrasound, The First Affiliated Hospital, Jiangxi Medical College, Nanchang University, Nanchang, China; 2 School of Advanced Manufacturing, Nanchang University, Nanchang, China

**Keywords:** High-frame-rate, Contrast-enhanced ultrasound, Clear cell renal cell carcinoma, Non-clear cell renal cell carcinoma, Blood vascular morphology, Computed tomography

## Abstract

**Background:**

Different subtypes of Renal Cell Carcinoma (RCC) exhibit distinct tumor vascular morphological features. However, current imaging techniques still have limitations in delineating the vascular morphology of RCC.

**Objective:**

The study aims to evaluate the value of high-frame-rate contrast-enhanced ultrasound (H-CEUS) in assessing the vascular morphology of RCC.

**Methods:**

A retrospective analysis was conducted on 163 RCC patients who were classified postoperatively into clear cell RCC (ccRCC) and non-clear cell RCC (nccRCC) groups. All patients underwent conventional US, conventional CEUS (C-CEUS), and H-CEUS preoperatively. Vascular morphology during the early phase of CEUS perfusion was categorized into five types (I-V). Differences in vascular morphology were compared using the *χ^2^* test or Fisher's exact test, and inter-observer agreement was evaluated using the Kappa coefficient.

**Results and discussion:**

A significant difference in CDFI was observed between the ccRCC and nccRCC groups (*χ^2^*=11.755, *P* = 0.0088). In C-CEUS and H-CEUS, type III vascular morphology was most common in ccRCC. The proportion was significantly higher in H-CEUS than in C-CEUS (62.6% *vs*. 45.8%; *χ^2^*=6.099, *P* = 0.014). Type IV was the most common vascular morphology in nccRCC, with no significant difference between C-CEUS (58.9%) and H-CEUS (44.6%) (*χ^2^*=2.289, *P* = 0.130). H-CEUS revealed significant vascular morphological differences in ccRCC ≤4 cm (*χ^2^*=9.307, *P* = 0.038), but not in nccRCC ≤4 cm or in any tumors >4 cm. Inter-observer agreement for vascular morphology evaluation was substantial for both C-CEUS (*κ*=0.751) and H-CEUS (*κ*=0.657) (both *P*<0.001).

**Conclusion:**

H-CEUS offers superior visualization of vascular morphology in ccRCC lesions ≤4 cm, providing valuable insights into its potential as a non-invasive imaging modality for differentiating RCC subtypes and tailoring treatment strategies.

## 1 INTRODUCTION

Renal Cell Carcinoma (RCC) is one of the most common malignant tumors of the urinary system, second only to bladder cancer in incidence. According to the latest statistics from the World Health Organization, renal carcinoma ranks 13^th^ among cancer-related deaths. With the widespread application and advancement of imaging techniques such as ultrasound, Computed Tomography (CT), and Magnetic Resonance Imaging (MRI), its detection has been increasing [1]. Based on biological behavior and pathogenesis, RCC can be classified into clear cell renal cell carcinoma (ccRCC) and non-clear cell renal cell carcinoma (nccRCC). Non-clear cell RCC primarily encompasses two common clinical subtypes: papillary Renal Cell Carcinoma (pRCC) and chromophobe Renal Cell Carcinoma (chRCC). Significant differences exist in the treatment and prognosis of RCC across various pathological subtypes. Thus, identifying RCC subtypes preoperatively is vital for devising optimal treatment strategies and is of significant importance in clinical management [2].

Although percutaneous renal tumor biopsy can provide histological diagnosis and is important for the evaluation and treatment of renal masses [3], it is an invasive procedure with a risk of bleeding, which limits its widespread use. Thus, preoperative non-invasive diagnosis of RCC subtypes is of importance. Routine ultrasound is the first-line modality for early screening of renal lesions. However, the detection rate for small tumors, lesions with unclear margins, or those with echogenicity similar to the renal parenchyma is still not satisfactory [4]. Additionally, routine ultrasound alone struggles to effectively identify RCC subtypes. Compared to other imaging modalities, Contrast-Enhanced Ultrasound (CEUS) offers unique advantages. It provides dynamic, continuous, and real-time imaging of microvascular blood flow, offering detailed information on tissue blood supply and clearly delineating tumor microvascular blood flow. Unlike CT and MRI, CEUS is a pure blood-pool angiographic technique that depends entirely on the contrast agent within blood vessels. Moreover, ultrasound contrast agents are well-tolerated and lack hepatotoxicity or nephrotoxicity. They will be eliminated *via* pulmonary exhalation, have a short half-life, and can be administered multiple times within a specified dosage range. These make CEUS a suitable imaging alternative for patients with chronic renal failure or renal impairment [1, 5]. CEUS is recommended for differentiating solid tumors from pseudorenal tumors, characterizing complex cysts and uncertain renal lesions, and monitoring the response to nonsurgical treatment of renal masses [6].

In traditional Contrast-Enhanced Ultrasound (C-CEUS), the rapid filling perfusion of renal lesions limits the acquisition of detailed arteriovenous perfusion information, thereby hindering the accurate diagnosis of solid renal cell carcinoma. However, high-frame-rate contrast-enhanced ultrasound (H-CEUS) addresses this limitation by increasing the frame acquisition frequency, enabling the evaluation of vascular enhancement with higher temporal and spatial resolution, particularly microvascular enhancement [7]. Previous studies have demonstrated that, compared to C-CEUS, H-CEUS offers better vascular visualization of focal liver lesions (FLL) during the arterial phase in patients with cirrhosis. This enhanced temporal resolution allows for the capture of fine perfusion details in the arterial phase [8]. In this study, C-CEUS and H-CEUS images from 163 RCC patients were retrospectively analyzed to assess the value of H-CEUS in visualizing RCC vascular morphology, aiming to provide additional diagnostic insights for accurate treatment planning.

## 2 METHODS

### 2.1 Objects of Study

A retrospective analysis was performed on 163 RCC patients at the First Affiliated Hospital of Nanchang University from January 2022 to November 2024, who underwent C-CEUS and H-CEUS examinations prior to surgery and received a histopathological diagnosis. This study was approved by the hospital ethics committee and the institutional review board (approval number: IIT2023174). Inclusion criteria included: 1) renal mass detected by routine ultrasound and contrast-enhanced ultrasound, 2) availability of histopathological results, and 3) no prior radiation, chemotherapy, or immunotherapy. Exclusion criteria included: 1) incomplete or unanalyzable contrast image data, 2) large tumors obscuring the normal renal cortex in the same section, 3) deep tumor location, patient obesity, or poor ultrasound penetration hindering image interpretation, and 4) cystic renal carcinoma.

### 2.2 Instruments and Methods

#### 2.2.1 Instrument

A Mindray Resona R9 ultrasonic diagnostic instrument equipped with an SC 5-1U probe operating at a frequency of 3-5 MHz was utilized. CEUS was performed using the Ultra-Wide Nonlinear imaging (UWN) technique. The frame rate for C-CEUS was 10 frames per second, while the frame rate for H-CEUS was 50-65 frames per second. Both modalities employed a low mechanical index of 0.06-0.08. SonoVue (Bracco, Italy), a sulfur hexafluoride microbubble-based contrast agent, was used. Prior to administration, 59 mg of SonoVue was added to 5 mL of 0.9% sodium chloride solution, and the mixture was thoroughly mixed to form a suspension.

### 2.3 Methods of Examination

The patient was positioned in a supine or lateral decubitus position. A routine ultrasound examination of tumors was performed to record the maximum diameter, laterality (left or right kidney), location (superior, middle, or inferior), composition (solid or cystic-solid), echogenicity (hyper-, iso-, or hypo-), shape (regular or irregular), boundary (clear or blurred), and presence or absence of calcification. The blood flow signals within the lesions were assessed using Color Doppler Flow Imaging (CDFI) and categorized into four grades: grade 0 (no blood flow), grade I (small amount of 1-2 starshaped blood flow), grade II (2-3 tar-shaped or short bundle blood flow), and grade III (rich blood flow with 3 or more color Doppler signals in a network or branching pattern). The maximum long-axis section of the lesion was visualized using conventional ultrasound. The C-CEUS mode was then activated, and 1.0 mL of SonoVue suspension was injected intravenously, followed by a rapid bolus of 5.0 mL of normal saline. Dynamic CEUS images were acquired for 1-3 minutes, and the early vascular morphology of solid portions of the CEUS image was recorded. A second angiography with H-CEUS was conducted at least 10 minutes after the first to ensure the contrast agent had fully cleared. During dynamic observation, the patient was instructed to breathe smoothly to avoid missing target lesions.

The imaging data were all stored in the ultrasound system. Two sonographers, each with over 5 years of CEUS experience, independently analyzed the images in a double-blind manner. Disagreements were resolved through joint discussion.

### 2.4 Analysis of Blood Vascular Morphology

The vascular morphology in the early stage of CEUS perfusion can be categorized into five distinct types: (I) Wheel-shaped, characterized by radial blood vessels emanating from the tumor center, (II) Peripheral vessels, featuring large vessels surrounding the tumor with sparse or absent intratumoral vessels, (III) Peripheral Nodule, defined by nodular enhancement around the tumor with dense, uniform distribution, (IV) Diffuse, presenting small, irregular vessels densely and evenly distributed within the lesion, and (V) Uncertain Shape, where vascular morphology is indistinct and does not conform to the other types. These vascular patterns, along with their schematic representations, are illustrated in Fig. (**1**).

### 2.5 Consistency Analysis

Two sonographers with over 5 years of CEUS experience (Physicians A and B) independently analyzed the vascular morphological characteristics of C-CEUS and H-CEUS images, blinded to patient clinical information and pathological findings. An observer-consistency test was used to assess agreement between the two physicians in evaluating vascular morphology.

### 2.6 Pathological Analysis

A pathologist with 10 years of experience in renal pathology re-examined all specimens, blinded to the clinical and imaging data, according to the fifth edition of the World Health Organization (WHO) classification of urogenital tumors [9]. Histopathological re-evaluation was also performed.

### 2.7 Statistical Analysis

Statistical analysis was performed using SPSS Statistics version 21.0 (IBM Corp, Armonk, NY, USA). Normally distributed measurement data were reported as mean ± Standard Deviation (SD), and independent *t*-tests were used for between-group comparisons. Categorical data were presented as frequencies and percentages, with comparisons between groups conducted using the *χ^2^* test or Fisher's exact test. Inter-rater reliability was assessed using Cohen's Kappa coefficient, with values interpreted as follows: 0-0.20, poor; 0.21-0.40, slight; 0.41-0.60, fair; 0.61-0.80, good; 0.81-1.00, very good. Statistical significance was set at *P* < 0.05.

## 3 RESULTS

### 3.1 Clinical Data and Conventional Ultrasound Features

Of the 163 patients with post-operatively histopathologically confirmed RCC, 107 were ccRCC and 56 were nccRCC. The nccRCC group included 21 cases of papillary RCC (10 type I and 11 type II), 19 cases of chromophobe RCC, 5 cases of Xp11.2 translocation/TFE3 gene fusion RCC, 4 cases of collecting duct carcinoma, 2 cases of mucinous tubular spindle cell carcinoma, and 5 cases of other rare RCC subtypes. The ccRCC group exhibited male predominance (62.6%, 67/107) with a mean age of 57.36±12.73 years (range: 28-85 years). The nccRCC group consistently exhibited male predominance (67.9%, 38/56) with a mean age of 53.3±15.14 years (range: 25-83 years). No significant differences were observed in gender or age between the ccRCC and nccRCC groups (both *P*> 0.05).

Comparison of the US between the ccRCC and nccRCC groups revealed distinct patterns in CDFI. ccRCC tumors predominantly exhibited grades II and III (31.8% and 22.9%, respectively), whereas nccRCC tumors were more frequently classified as grades 0 and I (21.4% and 44.6%, respectively). The difference was statistically significant (*χ^2^*=11.755, *P* = 0.008). Other ultrasound parameters, including nodule laterality, location, diameter, component, echogenicity, boundary, shape, and calcification, showed no significant differences (all *P*> 0.05) (Table **1**).

### 3.2 Comparison of C-CEUS and H-CEUS to Display Vascular Morphology in Different Sizes and Types of RCC

In both C-CEUS and H-CEUS, the primary manifestation of ccRCC was peripheral nodular enhancement (type III), which accounted for 45.8% (49/107) and 62.6% (67/107) of cases, respectively. This difference was statistically significant (*χ^2^*=6.099, *P* = 0.014). Conversely, nccRCC was primarily characterized by a diffuse enhancement pattern (type IV) in both C-CEUS and H-CEUS, observed 58.9% (33/56) and 44.6% (25/56) of cases, respectively. This difference was not statistically significant (*χ^2^* = 2.289, P = 0.130). Among tumors with a diameter ≤4 cm, the early perfusion vascular morphology of ccRCC on C-CEUS varied: 26 cases exhibited type III, 15 type IV, and 14 type V. In contrast, H-CEUS showed a different distribution, with 36 cases of type III, 8 of type IV, and 6 of type V, constituting a statistically significant difference (*χ^2^*=9.307, *P* = 0.038) (Fig. **2**). For nccRCC nodules ≤4 cm, no significant difference in vascular morphology was observed between C-CEUS and H-CEUS (*P*>0.05) (Fig. **3**). Moreover, in nodules >4 cm, no significant differences were found for either ccRCC or nccRCC between H-CEUS and C-CEUS (*P*>0.05) (Table **2**).

### 3.3 Consistency in the Evaluation of Vascular Morphology between Physician A and Physician B

Kappa coefficients of consistency evaluation results for 163 RCC perfusion patients between physician A and physician B were 0.751 (*P*<0.001) in C-CEUS and 0.657 (*P*<0.001) in H-CEUS (Table **3**).

## 4 DISCUSSION

RCC, the most prevalent primary renal malignancy, typically necessitates surgical resection. Clear cell renal cell carcinoma, constituting 70-80% of all RCC cases [10], is known for its aggressive nature and higher metastatic potential compared to other subtypes. In clinical practice, nccRCC is a collective term for RCC subtypes excluding ccRCC. Among nccRCC subtypes, papillary RCC (pRCC) and chromophobe RCC (chRCC) are the most common, accounting for 7-15% and 5-10% of all cases, respectively. These two subtypes are often considered independently for treatment planning and prognostic assessment. It is worth noting that the five-year survival rate of nccRCC is significantly better than that of ccRCC [11]. The primary clinical treatment options for RCC involve local or radical nephrectomy. In view of its abundant blood supply, radical nephrectomy is usually more suitable for ccRCC, while nccRCC is characterized by poor angiogenesis, lower malignancy, and superior prognosis than ccRCC, and is usually treated by local resection. However, except for patients with distant metastasis, they have a poorer prognosis, shorter overall survival, and higher mortality rates than ccRCC patients [12]. Given the significant differences in treatment and prognosis among RCC subtypes, accurately differentiating benign or malignant renal space-occupying lesions and identifying RCC subtypes have become crucial focuses in imaging research.

Routine ultrasound can offer details on the location, size, shape, boundary, and internal echo features of lesions [13]. However, it has limitations in visualizing the fine internal structure of tumors, which may lead to misdiagnosis of small lesions. The study found no statistically significant differences in position, composition, echo, boundary, shape, or diameter of nodules between the ccRCC and nccRCC groups (*P*>0.05). This indicates an overlap in the imaging appearance of these two subtypes, making it difficult to accurately identify RCC subtypes based solely on gray-scale ultrasound. Additional imaging modalities need to be integrated for differential diagnosis.

CDFI and Pulse Doppler can provide information on blood flow signals, arterial velocity, and resistance index within lesions, which are generally considered valuable for distinguishing between benign and malignant lesions. In this study, significant differences in CDFI were observed between ccRCC and nccRCC groups (χ^2^=11.755, *P* = 0.008). This also indicates that there are essential differences in hemodynamic characteristics between them. ccRCC is typically hypervascular, characterized by high microvessel density, arborizing vascular patterns, and enlarged vessel diameters. On the contrary, nccRCC is generally hypovascular and exhibits comparatively diminished blood perfusion. However, CDFI also has limitations in visualizing small vessels and low-velocity blood flow, and the reproducibility of blood flow displays can vary significantly across different instruments. While CDFI can provide some reference for assessing the characteristics of benign and malignant renal masses [14], it is not robust in differentiating RCC subtypes and lacks reliable ultrasound features for distinction. Further integration with other imaging techniques is needed for diagnostic evaluation.

CEUS, compared to CDFI, can provide detailed information about the distribution of blood vessels within a tumor, significantly improving the sensitivity of blood flow detection. CEUS demonstrated superior diagnostic performance over CDFI for renal tumors, achieving higher AUC (0.788 *vs*. 0.695), sensitivity (0.984 *vs*. 0.554), and accuracy (0.920 *vs*. 0.600) in distinguishing malignant from benign lesions [15]. Jiang *et al*. [16] found a strong correlation between CEUS features and tumor size and pathological characteristics in RCC, exceeding that between enhanced CT and tumor blood vessels. Moreover, studies have shown a strong correlation between ultrasound and CT/MRI in monitoring kidney tumor size [17]. Liu *et al*. [18] analyzed 97 quantitative CEUS parameters and found that CEUS could differentiate between ccRCC and nccRCC. Huang *et al*. [19] suggested that combining conventional US with CEUS features could help distinguish invasive from non-invasive RCC, offering potential preoperative predictive value for RCC invasiveness.

However, C-CEUS may encounter limitations in visualizing blood-rich lesions, especially small ones. The rapid arterial phase perfusion makes it challenging to clearly depict these details at the standard frame rate of 9-15 fps, which may affect diagnostic accuracy. H-CEUS addresses this limitation by increasing the frame rate to over 50-70 fps, thereby achieving higher temporal resolution than C-CEUS. This enhanced resolution allows for a more detailed visualization of the arterial phase [20], enabling the observation of detailed blood perfusion patterns in small, highly vascularized lesions. Previous studies have also demonstrated that, compared with C-CEUS, H-CEUS can accurately depict the early arterial perfusion characteristics of focal liver lesions (FLL), particularly their vascular morphology [21]. Based on these findings, we aim to utilize H-CEUS to provide valuable insights into the vascular perfusion patterns in the arterial phase of renal cell carcinoma.

C-CEUS may not provide a detailed representation of blood perfusion in small lesions with very rapid arterial perfusion. H-CEUS employed in this study significantly enhances temporal resolution, enabling the maximum capture of relevant information from contrast agent microbubbles. This improvement substantially enhances the clarity of visualization of tiny vascular structures. In this study, under C-CEUS and H-CEUS, the predominant manifestation in ccRCC patients was type III peripheral nodular enhancement, accounting for 45.8% (49/107) and 62.6% (67/107), respectively. In contrast, the primary manifestation in nccRCC patients was type IV diffuse enhancement, with 58.9% (33/56) and 44.6% (25/56), respectively. This difference highlights the distinct vascular composition of the two renal tumor subtypes. ccRCC typically presents with a dense network of new blood vessels, characterized by larger diameters. After forming arteriovenous fistulas with normal vessels, the lesion receives an abundant blood supply. Moreover, due to rapid tumor growth, ccRCC is prone to necrosis, cystic changes, or bleeding, leading to the formation of cube-shaped or columnar cancer cells arranged in sheets or nests around the lesion. This arrangement results in peripheral nodular enhancement under CEUS [22]. On the other hand, nccRCC is typically a tumor with a deficient blood supply, sparse internal blood vessels, small vessel diameters, and low density. The contrast agent enters the tumor slowly, resulting in low enhancement, sparse filling, and a diffuse enhancement pattern of blood vessels [23].

Some studies have suggested that lesion size can influence the CEUS visualization of vascular distribution during the arterial phase [24]. Liu *et al*. proposed that the imaging findings of RCC are largely dependent on the pathological characteristics and lesion size, with differences in blood vascular morphology between RCC lesions greater than 4 cm and those less than or equal to 4 cm, as well as variations in the degree of compression of adjacent normal renal parenchyma and the number of arteriovenous fistulas within the mass [25]. However, the clinical utility of H-CEUS in small kidney lesions remains to be further explored. Therefore, this study aimed to compare and analyze the differences between H-CEUS and C-CEUS in lesions larger than 4 cm and those 4 cm or smaller.

The results of this study demonstrated that among nodules with a diameter ≤4 cm, the early perfusion vascular morphology of the ccRCC group under C-CEUS showed the following distribution: 0 cases of type I, 3 of type II, 26 of type III, 15 of type IV, and 14 of type V. Under H-CEUS, the morphology was distributed as follows: type I in 1 case, type II in 7, type III in 36, type IV in 8, and type V in 6. The difference between C-CEUS and H-CEUS was statistically significant. This discrepancy can be attributed to the pathological characteristics of ccRCC. It is predominantly a hematogenous tumor with large cancer nests, abundant and thick blood vessels, and frequent arteriovenous fistulas observed microscopically. The tumor vessels are abundant and dense, allowing contrast agents to rapidly and extensively infiltrate the tumor. In small lesions, peripheral nodular blood vessels are often smaller, and rapid blood perfusion can cause them to flash by quickly, which C-CEUS frame rates may not accurately capture. However, by increasing the CEUS frame rate, H-CEUS can more clearly visualize these typical nodular blood vessels [26]. Consequently, H-CEUS mode exhibits a significant increase in the display of type III blood vascular morphology. Additionally, due to the small size of the lesion and limited internal vascular spaces, rapid blood perfusion can hinder the capture of the initial location of microbubbles within the lesion using C-CEUS. This accounts for the fewer type I and type II vascular morphology cases observed with C-CEUS compared to H-CEUS. Moreover, the small lesion size leads to a finer distribution of blood vessels and faster perfusion times, which may cause misclassification in C-CEUS. Among the 58 lesions in the ccRCC group with a nodule diameter ≤4 cm, 14 lesions initially classified as type V under C-CEUS were reclassified into other types using H-CEUS. H-CEUS, with its higher frame rate and superior temporal resolution, provides excellent visualization of focal arterial perfusion patterns and vascular morphology, enabling clearer depiction of vascular morphology in RCC patients during the early perfusion stage [27].

Consistent with previous studies [28], H-CEUS has a higher temporal resolution than C-CEUS, enabling better depiction of real-time changes in the arterial phase. Particularly in renal cell carcinoma with a maximum diameter of ≤4 cm, H-CEUS can help visualize arterial perfusion patterns and vascular morphology, mitigating the limitations of C-CEUS.

In the nccRCC group with tumor diameters ≤4 cm, no significant differences in vascular morphology were observed between C-CEUS and H-CEUS. This may be related to the fact that nccRCC is primarily characterized by hypovascularity, sparse distribution of microvessels within the tumor, and limited maximum uptake of contrast agent. This also explains the predominance of type IV vascular morphology observed in both C-CEUS and H-CEUS. Similarly, among lesions >4 cm in diameter, no significant differences in vascular morphology were detected between H-CEUS and C-CEUS for either ccRCC or nccRCC. This may be attributed to the increased time required for microcirculation perfusion as the lesion volume grows. C-CEUS is sufficient to capture the perfusion process and vascular structure of the blood. It is worth noting that discrepancies in vascular morphology classification were observed between C-CEUS and H-CEUS in the nccRCC group. Specifically, among lesions with a diameter ≤4 cm, 6 cases initially classified as type IV on C-CEUS were reassessed by H-CEUS into type I (n=4) and type II (n=2). Furthermore, in lesions >4 cm, 2 cases showing type IV on C-CEUS were reclassified by H-CEUS as type I (n=1) and type II (n=1). The higher resolution of H-CEUS enables clearer visualization of the microvasculature, revealing specific vascular structural features that were not distinctly shown on C-CEUS. This capability allows cases initially classified as the diffuse enhancement pattern (Type IV) on C-CEUS to be further differentiated into more specific Type I or Type II on H-CEUS. This reclassification phenomenon not only refines diagnostic classification but also reduces the number of cases categorized as the non-specific “diffuse” type.

In this study, the frame rate for C-CEUS was set at 10 fps, while H-CEUS utilized a higher frame rate ranging from 50 to 65 fps. This significant increase in frame rate greatly enhanced the temporal resolution of H-CEUS images. The results showed that H-CEUS provided better visualization of RCC vasculature than C-CEUS, especially in the small nodule group. This advantage mainly stemmed from the significantly enhanced temporal resolution of H-CEUS, which enabled clearer depiction of RCC vascular morphology.

Currently, there is no standardized method for interpreting RCC vascular morphology, leading to variability among observers. Therefore, to ensure the reliability of the subjective interpretation of vascular morphology during perfusion, high interobserver consistency is crucial. To this end, this study further assessed interobserver consistency between physicians A and B in the display of vascular morphology under H-CEUS and C-CEUS. The results showed that when assessing the consistency of vascular morphology observations in 163 RCC cases under C-CEUS mode, the Kappa coefficient was 0.751 (P<0.001). Similarly, under H-CEUS, the Kappa coefficient was 0.657 (P<0.001). The Kappa coefficient for H-CEUS was slightly lower than that for C-CEUS (0.657 *vs*. 0.751). This discrepancy may be due to the fact that, under C-CEUS, the number of cases in which physicians A and B could not determine the vascular morphology (Type IV + Type V) was significantly higher than under H-CEUS. Specifically, for physician A, 55.24% ((55+39)/163) of cases under the C-CEUS exhibited indeterminate vascular morphology, a proportion significantly higher than that under the H-CEUS mode ((36+23)/163) (*P*<0.001). Similarly, for physician B, 54.2% ((54+33)/163) of cases under the C-CEUS had indeterminate vascular morphology, which was also significantly higher than that under the H-CEUS mode (42.33% ((40+29)/163)) (*P* = 0.046). Despite these differences, the H-CEUS mode still demonstrated a high level of inter-observer consistency and exhibited good clinical application value. Therefore, H-CEUS is anticipated to be an effective and accurate novel technique for assessing vascular morphology in the early stage of perfusion of CEUS in RCC.

## 5 STUDY LIMITATION

This study has several limitations that should be acknowledged. Firstly, the analysis in this study was limited to the differences in vascular perfusion morphology between ccRCC and nccRCC during the early arterial phase, excluding the differences in contrast patterns between the two groups during the regression phase. Secondly, as a single-center retrospective study with a relatively small sample size, further validation through prospective multicenter studies is needed to corroborate the early vascular morphology findings of RCC in this study.

## CONCLUSION

H-CEUS, with its superior frame rate, enables more precise tracking of microbubble movement and detailed observation of microvascular perfusion. This enhanced temporal resolution allows for a clearer visualization of focal perfusion compared to C-CEUS. By improving temporal resolution, H-CEUS can effectively display vascular morphology during the early stages of perfusion in ccRCC less than 4 cm.

## Figures and Tables

**Fig. (1):**
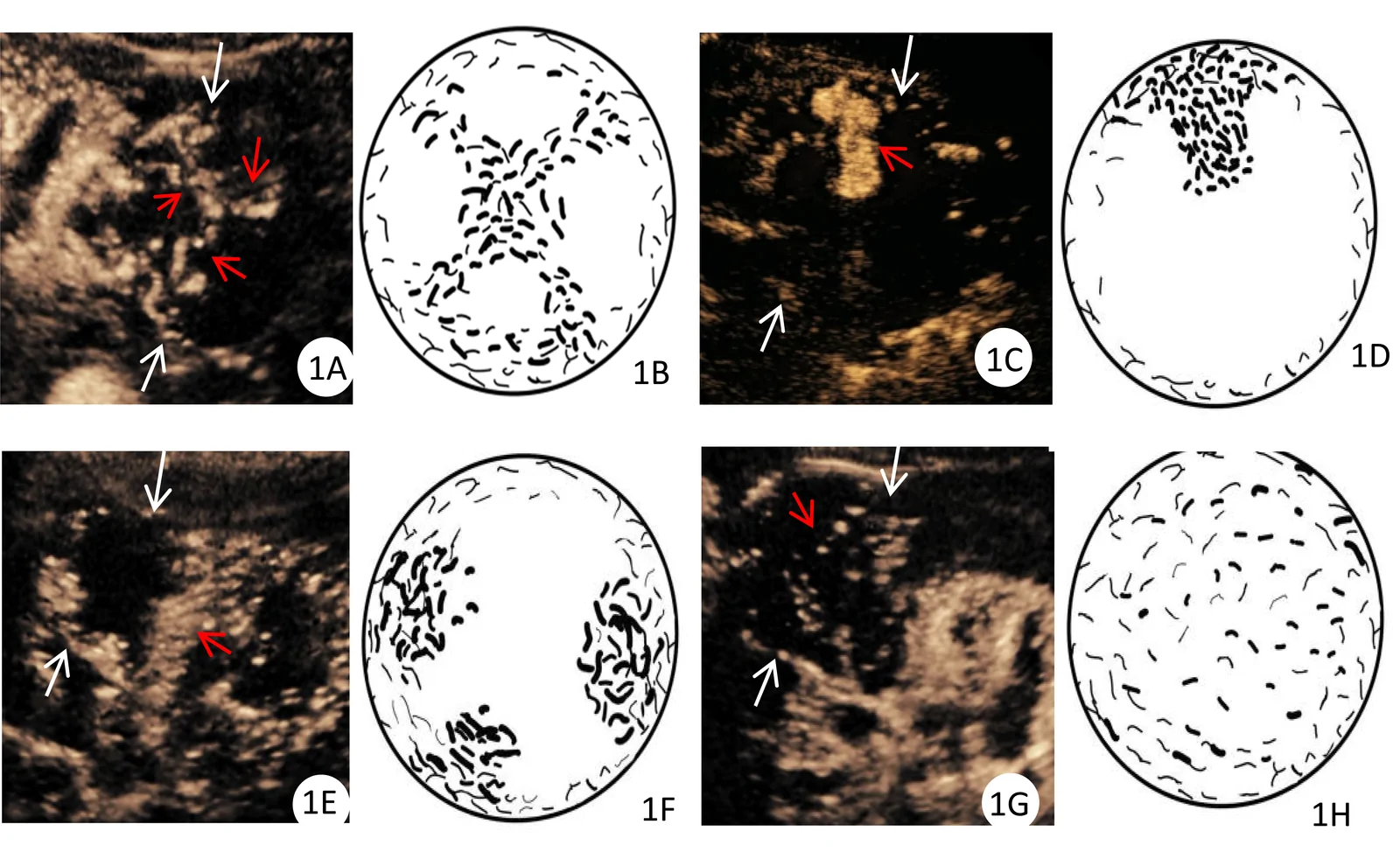
Comparison of vascular morphology in renal cell carcinoma: contrast-enhanced ultrasound (CEUS) images and schematic diagrams. (**1A**) and (**1B**) demonstrate wheel-shaped enhancement (Type I) on CEUS (**1A**) and its corresponding schematic diagram (**1B**), characterized by wheel-like vessels within the tumor during the early perfusion phase (red arrows: wheel-shaped vessels; white arrow: peritumoral area). (**1C**) and (**1D**) display peripheral vessel enhancement (Type II) on CEUS (**1C**) and the schematic (**1D**), showing enhanced peripheral vasculature in the early perfusion stage (red arrow: peripheral vessels; white arrow: peritumoral area). (**1E**) and (**1F**) illustrate peripheral nodular enhancement (Type III) on CEUS (**1E**) and the schematic (**1F**), where nodular enhancement is observed along the tumor periphery in early perfusion (red arrows: nodules; white arrow: peritumoral area). (**1G**) and (**1H**) present diffuse enhancement (Type IV) on CEUS (**1G**) and the schematic (**1H**), with enhancement both inside and surrounding the tumor during early perfusion (red arrow: intratumoral area; white arrow: peritumoral area).

**Fig. (2):**
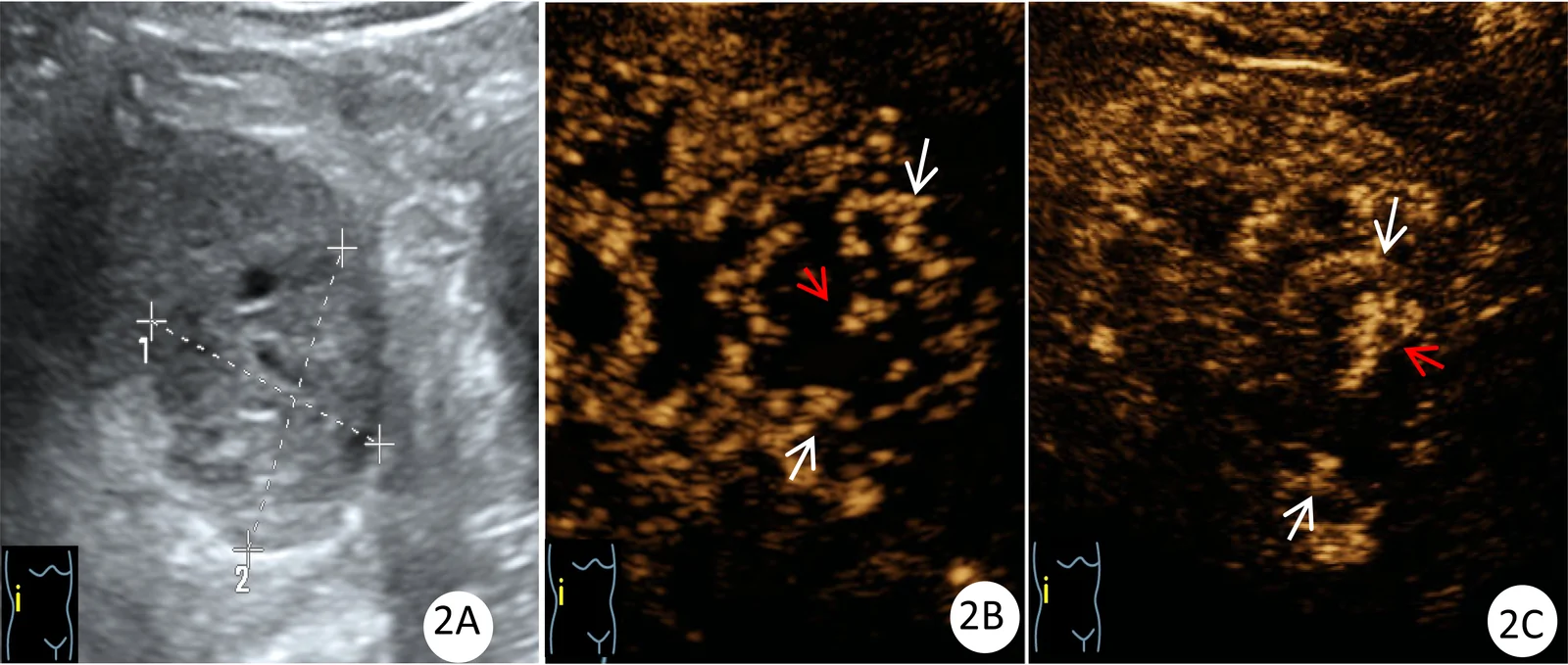
Vascular morphology in ccRCC on C-CEUS and H-CEUS. (**2A**) shows a 3.5cm hypoechoic mass at the lower pole of the right kidney, diagnosed as ccRCC. (**2B**) illustrates that, in the early perfusion phase of C-CEUS, the lesion exhibits a diffuse enhancement pattern (Type IV) in both the periphery and the center (red arrow indicates the intratumoral area; white arrow indicates the peritumoral area). (**2C**) shows that in the early perfusion phase of H-CEUS, the lesion demonstrates peripheral nodular enhancement (Type III) (red arrow: peripheral enhancing nodules; white arrow: peritumoral area)

**Fig. (3):**
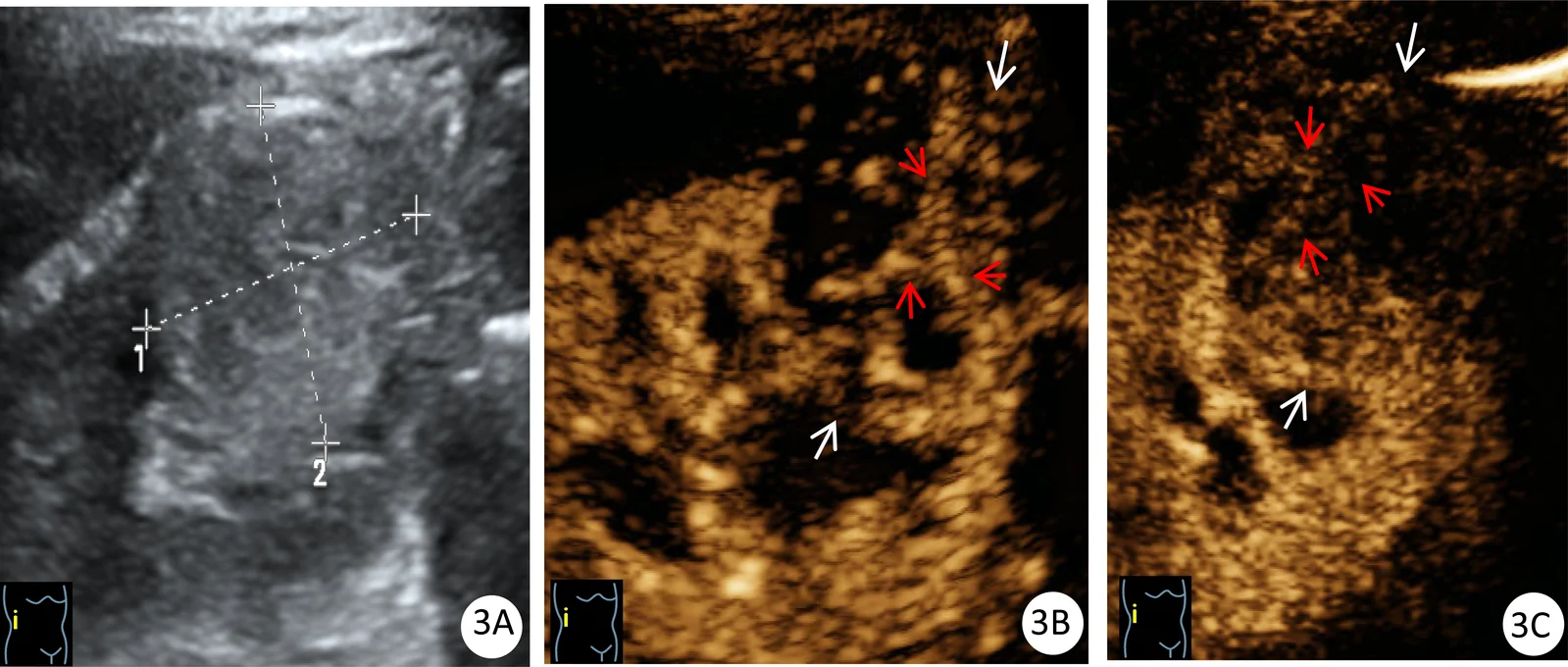
Vascular morphology in nccRCC on C-CEUS and H-CEUS. (**3A**) shows a 3.4-cm hyperechoic mass at the middle pole of the right kidney that was diagnosed as papillary RCC. (**3B)** and (**3C)** show that in the early perfusion stage, both C-CEUS (**3B**) and H-CEUS (**3C**) demonstrate wheel-shaped enhancement (Type I) within the tumor (red arrow: wheel-shaped vessels; white arrow: peritumoral area).

**Table 1:** Characteristics of clinical parameters and conventional US in ccRCC and nccRCC.

		**ccRCC (n=107)**	**nccRCC (n=56)**	***χ^2^* or *t***	***p*-value**
Gender, n(%)	Male	67(62.6%)	38(67.9%)	0.440	0.606
	Female	40(37.4%)	18(32.1%)		
Age(years): mean±SD		57.36±12.73	53.3±15.14	1.806	0.073
Laterality, n(%)	Left	55(51.4%)	34(60.7%)	1.286	0.321
	Right	52(48.6%)	22(39.3%)		
Location, n(%)	Superior	41(38.3%)	13(23.2%)	3.828	0.146
	Middle	34(31.8%)	23(41.1%)		
	Inferior	32(29.9%)	20(35.7%)		
Component, n(%)	Solid	92(86.0%)	49(87.5%)	0.073	0.816
	Cystic-solid	15(14.0%)	7(12.5%)		
Echogenicity, (n/%)	Hyper-	31(29.0%)	18(32.1%)	1.825	0.404
	Iso-	2(1.9%)	3(5.4%)		
	Hypo-	74(69.1%)	35(62.5%)		
Boundary, n(%)	Clear	105(98.1%)	52(92.9%)	2.884	0.182
	Blurred	2(1.9%)	4(7.1%)		
Shape, n(%)	Regular	103(96.3%)	51(91.1%)	1.898	0.277
	Irregular	4(3.7%)	5(8.9%)		
CDFI, n(%)	0	13(12.1%)	12(21.4%)	11.755	0.008
	I	28(26.2%)	25(44.6%)		
	II	34(31.8%)	8(14.3%)		
	III	32(29.9%)	11(19.6%)		
Calcification, n(%)	Present	3(2.8%)	5(8.9%)	2.955	0.125
	Absent	104(97.2%)	51(91.1%)		
Tumor diameter (cm): mean±SD		4.35±2.21	4.65±2.16	-0.834	0.405

**Table 2:** Comparison of vascular morphology between C-CEUS and H-CEUS in different sizes of CCRCC and nccRCC.

**Type**	**CEUS**	**Wheel-shaped**	**Peripheral vessels**	**Peripheral nodule**	**Diffuse**	**Unidentified**	***χ^2^***	***P***
≤4cm ccRCC(n=58)	C-CEUS	0(0.0%)	3(5.2%)	26(44.8%)	15(25.9%)	14(24.1%)	9.307	0.038
	H-CEUS	1(1.7%)	7(12.1%)	36(62.1%)	8(13.8%)	6(10.3%)		
>4cm ccRCC(n=49)	C-CEUS	1(2.0%)	5(10.2%)	23(46.9%)	7(14.3%)	13(26.5%)	4.245	0.365
	H-CEUS	1(2.0%)	6(12.2%)	31(63.3%)	3(6.1%)	8(16.3%)		
≤4cm nccRCC(n=29)	C-CEUS	5(17.2%)	1(3.4%)	0(0.0%)	17(58.6%)	6(20.7%)	6.069	0.114
	H-CEUS	11(37.9%)	4(13.8%)	0(0.0%)	11(37.9%)	3(10.3%)		
>4cm nccRCC(n=27)	C-CEUS	3(11.1%)	2(7.4%)	0(0.0%)	16(59.3%)	6(22.2%)	0.639	0.948
	H-CEUS	4(14.8%)	3(11.1%)	0(0.0%)	14(51.9%)	6(22.2%)		

**Table 3:** Analysis of the consistency of the evaluation results of RCC vascular morphology between physician A and physician B.

**Physician B**	**Physician A**					**Total**
	**Wheel-** **shaped**	**Peripheral vessels**	**Peripheral nodule**	**Diffuse**	**Unidentified**	
C-CEUS						
Wheel- shaped	7(4.3%)	1(0.6%)	2(1.2%)	1(0.6%)	1(0.6%)	12(7.4%)
Peripheral vessels	0(0.0%)	8(4.9%)	3(1.8%)	2(1.2%)	1(0.6%)	14(8.6%)
Peripheral nodule	0(0.0%)	1(0.6%)	42(25.8%)	4(2.5%)	3(1.8%)	50(30.7%)
Diffuse	2(1.2%)	1(0.6%)	1(0.6%)	46(28.2%)	4(2.5%)	54(33.1%)
Unidentified	0(0.0%)	0(0.0%)	1(0.6%)	2(1.2%)	30(18.4%)	33(20.2%)
Total	9(5.5%)	11(6.7%)	49(30.1%)	55(33.7%)	39(23.9%)	163(100.0%)
H-CEUS						
Wheel- shaped	13(8.0%)	1(0.6%)	3(1.8%)	1(0.6%)	1(0.6%)	19(11.7%)
Peripheral vessels	1(0.6%)	12(7.4%)	3(1.8%)	2(1.2%)	0(0.0%)	18(11.0%)
Peripheral nodule	1(0.6%)	2(1.2%)	50(30.7%)	3(1.8%)	1(0.6%)	57(35.0%)
Diffuse	1(0.6%)	3(1.8%)	5(3.1%)	28(17.2%)	3(1.8%)	40(24.5%)
Unidentified	1(0.6%)	2(1.2%)	6(3.7%)	2(1.2%)	18(11.1%)	29(17.8%)
Total	17(10.4%)	20(12.3%)	67(41.1%)	36(22.1%)	23(14.1%)	163(100.0%)

## Data Availability

The data of current study are available from corresponding author, [W.Z], on a reasonable request.

## LIST OF ABBREVIATIONS

RCC: Renal Cell Carcinoma
H-CEUS: High-Frame-Rate Contrast-Enhanced Ultrasound
nccRCC: Non-Clear Cell Renal Cell Carcinoma
C-CEUS: Conventional Contrast-Enhanced Ultrasound
CT: Computed Tomography
MRI: Magnetic Resonance Imaging
pRCC: Papillary Renal Cell Carcinoma
UWN: Ultra-Wide Nonlinear Imaging
FLL: Focal Liver Lesions
CDFI: Color Doppler Flow Imaging
WHO: World Health Organization
SD: Standard Deviation

## STANDARDS OF REPORTING

STROBE guidelines were followed.

## References

[1] Zhao P., Zhu J., Wang L., Li N., Zhang X., Li J., Luo Y., Li Q. (2023). Comparative diagnostic performance of contrast-enhanced ultrasound and dynamic contrast-enhanced magnetic resonance imaging for differentiating clear cell and non-clear cell renal cell carcinoma.. Eur. Radiol..

[2] Bahadoram S., Davoodi M., Hassanzadeh S., Bahadoram M., Barahman M., Mafakher L. (2022). Renal cell carcinoma: An overview of the epidemiology, diagnosis, and treatment.. G. Ital. Nefrol..

[3] Patel H.D., Johnson M.H., Pierorazio P.M., Sozio S.M., Sharma R., Iyoha E., Bass E.B., Allaf M.E. (2016). Diagnostic accuracy and risks of biopsy in the diagnosis of a renal mass suspicious for localized renal cell carcinoma: Systematic review of the literature.. J. Urol..

[4] Wylie B., Necas M., Heaney A. (2020). Visualisation of focal renal lesions on ultrasound: A review of 518 lesions with contrast CT correlation.. Australas. J. Ultrasound Med..

[5] Stock K.F., Slotta-Huspenina J., Kübler H., Autenrieth M. (2019). Innovative Ultraschalldiagnostik bei Nierentumoren.. Urologe A.

[6] Sidhu PS, Cantisani V, Dietrich CF (2022). Prostate cancer review: Genetics, diagnosis, treatment options, and alternative approaches.. Molecules.

[7] Zhu J., Lin S., Leow C.H., Rowland E.M., Riemer K., Harput S., Weinberg P.D., Tang M.X. (2019). High frame rate contrast-enhanced ultrasound imaging for slow lymphatic flow: Influence of ultrasound pressure and flow rate on bubble disruption and image persistence.. Ultrasound Med. Biol..

[8] Giangregorio F., Garolfi M., Mosconi E., Ricevuti L., Debellis M.G., Mendozza M., Esposito C., Vigotti E., Cadei D., Abruzzese D. (2022). High frame-rate contrast enhanced ultrasound (HIFR-CEUS) in the characterization of small hepatic lesions in cirrhotic patients.. J. Ultrasound.

[9] Moch H., Amin M.B., Berney D.M., Compérat E.M., Gill A.J., Hartmann A., Menon S., Raspollini M.R., Rubin M.A., Srigley J.R., Hoon Tan P., Tickoo S.K., Tsuzuki T., Turajlic S., Cree I., Netto G.J. (2022). The 2022 World Health Organization Classification of tumours of the urinary system and male genital organs—Part A: Renal, penile, and testicular tumours.. Eur. Urol..

[10] Vermassen T., De Meulenaere A., Van de Walle M., Rottey S. (2017). Therapeutic approaches in clear cell and non-clear cell renal cell carcinoma.. Acta Clin. Belg..

[11] Deng J., Li L., Xia H., Guo J., Wu X., Yang X., Hong Y., Chen Q., Hu J. (2019). A comparison of the prognosis of papillary and clear cell renal cell carcinoma.. Medicine (Baltimore).

[12] Steffens S, Roos FC, Janssen M (2014). Clinical behavior of chromophobe renal cell carcinoma is less aggressive than that of clear cell renal cell carcinoma, independent of Fuhrman grade or tumor size.. Prostate.

[13] Yamauchi F.I., Paiva O.A., Mussi T.C., Francisco M.J., Baroni R.H. (2020). A comparative study of ultrasound and cross-sectional imaging for detection of small renal masses: Anatomic factors and radiologist’s experience.. Einstein (Sao Paulo).

[14] Mao Y., Mu J., Zhao J., Yang F., Zhao L. (2022). The comparative study of color doppler flow imaging, superb microvascular imaging, contrast-enhanced ultrasound micro flow imaging in blood flow analysis of solid renal mass.. Cancer Imaging.

[15] Li C., Qi L., Geng C., Xiao H., Wei X., Zhang T., Zhang Z., Wei X. (2025). Comparative diagnostic performance of color doppler flow imaging, microflow imaging and contrast-enhanced ultrasound in solid renal tumors.. Acad. Radiol..

[16] Jiang J., Chen Y., Zhou Y., Zhang H. (2010). Clear cell renal cell carcinoma: Contrast-enhanced ultrasound features relation to tumor size.. Eur. J. Radiol..

[17] Kim S.S.Y., Breau R., Mallick R., Kapoor A., Finelli A., Pouliot F., Tanguay S., Fairey A., Bhindi B., Lavallee L., So A., Rendon R. (2022). Ultrasound correlates highly with cross sectional imaging for small renal masses in a contemporary cohort.. Urology.

[18] Liu H., Cao H., Chen L., Fang L., Liu Y., Zhan J., Diao X., Chen Y. (2022). The quantitative evaluation of contrast-enhanced ultrasound in the differentiation of small renal cell carcinoma subtypes and angiomyolipoma.. Quant. Imaging Med. Surg..

[19] Huang X., Wang N., Liu L., Zhu J., Wang Z., Wang T., Nie F. (2023). Pre-operative prediction of invasiveness in renal cell carcinoma: The role of conventional ultrasound and contrast-enhanced ultrasound.. Ultrasound Med. Biol..

[20] Yang F., Jiang L., Ma Y., He Y., Xu Y., Yang R., Wu B., Li Y. (2024). Quantitative assessment of early kidney injury using high frame rate contrast-enhanced ultrasonography in a rabbit model of diabetic nephropathy.. Med. Ultrason..

[21] Fei X., Zhu L., Han P., Jiang B., Li M., Li N., Jiao Z., Clevert D.A., Luo Y. (2024). Value of high frame rate contrast-enhanced ultrasound in predicting microvascular invasion of hepatocellular carcinoma.. Insights Imaging.

[22] Dong X.Q., Shen Y., Xu L.W., Xu C.M., Bi W., Wang X.M. (2009). Contrast-enhanced ultrasound for detection and diagnosis of renal clear cell carcinoma.. Chin. Med. J. (Engl.).

[23] Zhang W., Wang J., Chen L., Shi J. (2024). Qualitative and quantitative assessment of non-clear cell renal cell carcinoma using contrast-enhanced ultrasound.. BMC Urol..

[24] Putz F.J., Verloh N., Erlmeier A., Schelker R.C., Schreyer A.G., Hautmann M.G., Stroszczynski C., Banas B., Jung E.M. (2019). Influence of limited examination conditions on contrast-enhanced sonography for characterising liver lesions.. Clin. Hemorheol. Microcirc..

[25] Liu Y., Kan Y., Zhang J., Li N., Wang Y. (2021). Characteristics of contrast‐enhanced ultrasound for diagnosis of solid clear cell renal cell carcinomas ≤4 cm: A meta‐analysis.. Cancer Med..

[26] Zhu J., Li N., Zhao P., Wang Y., Song Q., Song L., Li Q., Luo Y. (2023). Contrast‐enhanced ultrasound ( CEUS ) of benign and malignant renal tumors: Distinguishing CEUS features differ with tumor size.. Cancer Med..

[27] Wang J., Shi J., Gao L., Hu W., Chen M., Zhang W. (2024). High-frame-rate contrast-enhanced ultrasound to differentiate between clear cell renal cell carcinoma and angiomyolipoma.. BMC Cancer.

[28] Zhang W., Wang J., Chen L. (2024). Characteristics of high frame frequency contrast-enhanced ultrasound in renal tumors.. BMC Med. Imaging.

